# 

**Published:** 1909-04

**Authors:** 


					﻿Sixty־fourth Year.
Vol, LX,lVv	APRIL, 1909.	No. 9
BUFFALO
MEDICAL JOURNAL
Established 1845 by AUSTIN FLINT M. D.
^^77	־־־־ב
EDITOR:	1
WILLIAM WARREN POTTER, M. D. X
ASSISTANT EDITORS:	'׳׳׳־——־־
WILLIAM C. KRAUSS, M. D.	NELSON W. WILSON, M. D.
ASSOCIATE EDITORS:
JAMES WRIGHT PUTNAM, M. D. ERNEST WENDE, M. D.
JOHN PARMENTER, M. D.	JOHN A. MILLER, Ph. D.
MAUD J. FRYE, M. D.
				

